# Genomic language models with *k*-mer tokenization strategies for plant genome annotation and regulatory element strength prediction

**DOI:** 10.1007/s11103-025-01604-7

**Published:** 2025-07-31

**Authors:** Shosuke Suzuki, Kazumasa Horie, Toshiyuki Amagasa, Naoya Fukuda

**Affiliations:** 1 Faculty of Life and Environmental Sciences Tsukuba-Plant Innovation Research Center, University of Tsukuba, Tsukuba, Japan; 2 Center for Computational Sciences, University of Tsukuba, Tsukuba, Japan

**Keywords:** Dna, *k*-mer, Transformer, Genomic language model, Genome annotation

## Abstract

Recent advances in genomic language models have improved the accuracy of *in silico* analyses, yet many rely on resource-intensive architectures. In this study, we focus on the impact of *k*-mer tokenization strategies–specifically varying window sizes (three to eight) and overlap schemes–on the performance of transformer-based genomic language models. Through extensive evaluation across multiple plant genomic tasks, including splice site and alternative polyadenylation site prediction, we show that thoughtful design of the *k*-mer tokenizer plays a critical role in model performance, often outweighing model scale. In particular, overlap-based tokenization generally enhances performance by preserving local sequence context, while certain non-overlap configurations achieve competitive accuracy with improved computational efficiency in some tasks. Despite using a smaller model, our approach performs on par with the state-of-the-art AgroNT model in many cases. These results emphasize that *k*-mer tokenization, not merely model size, is a key determinant of success in genomic sequence modeling. Our findings provide practical guidance for designing efficient genomic language models tailored to plant biology.

## Key message

BERT models with optimized k-mer tokenization achieve comparable performance to larger models in plant genomic tasks, offering efficient alternatives for researchers with limited computational resources.

## Introduction

The field of plant genomics has rapidly evolved in the past two decades, fueled by dramatic advances in next-generation sequencing (NGS) technologies (Mardis 2008). A major milestone in this evolution was the publication of the *Arabidopsis thaliana* genome in 2000 (Initiative 2000), which laid the foundation for large-scale sequencing initiatives across various plant species. Since then, the increasing affordability and throughput of NGS have enabled the decoding of more than 200 complete plant genomes, along with hundreds of additional draft assemblies (Sun et al 2022; Kersey 2019). This explosive growth is also evident in centralized resources such as the NCBI Reference Sequence (RefSeq) database, which now includes nearly 600 plant genome assemblies with curated annotations, covering a wide range of taxonomic groups (Goldfarb et al 2024). These trends highlight the rapid expansion of publicly available genomic resources and underscore the need for scalable computational methods to analyze them effectively.

However, the assembly of a genome sequence is just the first step in the complex process of decoding plant genomes. The crucial task of structural and functional annotation, identifying and characterizing important genomic regions such as genes and regulatory elements, demands extensive additional experimentation and computational analysis. As the volume of genomic data continues to expand at an unprecedented rate, there is a growing need for computational methods, including machine learning, that can efficiently and accurately analyze and interpret this wealth of information (Angermueller et al 2016; Libbrecht and Noble 2015). This challenge is at the intersection of biology and computer science, necessitating innovative approaches to harness the full potential of plant genomic data.

Recent advances in artificial intelligence, particularly in large language models (LLMs), have opened new avenues for genomic analysis. Among these, the Genomic Language Model (gLM) or the DNA Foundation Model (DNA-FM), variants of LLM trained on genomic sequences using transformer architectures (Vaswani et al 2023) - such as DNABERT (Ji et al 2021), the Nucleotide Transformer (NT) (Dalla-Torre et al 2023) and the Agronomic Nucleotide Transformer (AgroNT) (Mendoza-Revilla et al 2024), have shown remarkable performance in various genetic prediction tasks by capturing complex patterns within large corpora of genomic sequence.

The application of gLMs promises to enhance the accuracy of *in silico* experiments and contribute to a deeper understanding of genetic mechanisms (Fishman et al 2023; Shmelev et al 2024). Notably, several prominent gLMs, including DNABERT and AgroNT, utilize *k*-mer tokenization strategies. Compared to vocabulary-learning methods like Byte-Pair Encoding (Sennrich et al 2016) or SentencePiece (Kudo and Richardson 2018), *k*-mer approaches are training-free and leverage fixed-length sequence units familiar in bioinformatics. However, the selection of key *k*-mer parameters, such as window size (*k*) and the overlap scheme between consecutive tokens, is often empirically driven rather than optimized for the characteristics of genomic sequences or specific downstream tasks. A more biologically informed design of *k*-mer tokenization may offer further performance gains. Meanwhile, large-scale models such as NT and AgroNT require significant computational resources (Zhang et al 2023a), limiting their accessibility and broader adoption in the research community.

This study aims to provide practical insight into the design of *k*-mer tokenizers for gLM by systematically analyzing the trade-offs between tokenization granularity, computational efficiency, and model performance. In particular, we investigate how the choice of *k*-mer size and token overlap affects not only prediction accuracy but also the number of generated tokens directly impacting model interpretability and computational cost. To this end, we train domain-specific models using various *k*-mer tokenization strategies and compare them against two gLMs that also employ *k*-mer tokenization: DNABERT, pre-trained on the human reference genome, and AgroNT, trained on genomes from 48 edible plant species. Our goal is to identify reasonable and effective tokenization strategies for transformer-based models in plant genomics, especially under computational resource constraints. By balancing efficiency, accuracy, and interpretability, our findings aim to support the broader adoption of gLMs in biological research and provide a practical foundation for scalable genomic language modeling in resource-constrained settings.

## Materials and methods

**Fig. 1:**
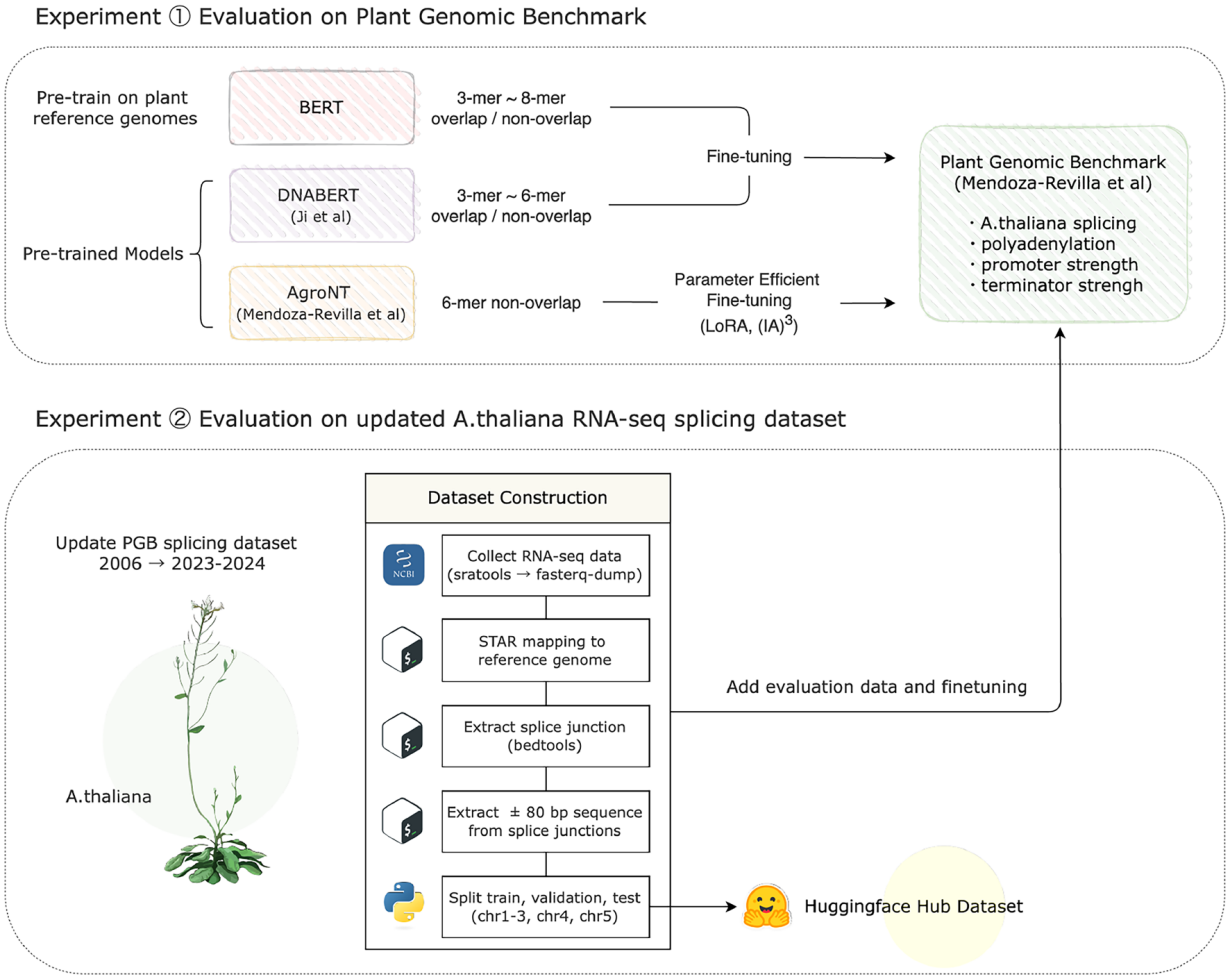
Overview of the k-mer tokenization strategies experiments in this study

Evaluation of the *k*-mer tokenization strategies was conducted on two types of datasets: (1) Plant Genomic Benchmarks (PMB) proposed in the Agronomic Nucleotide Transformer (AgroNT) paper (Mendoza-Revilla et al 2024), which include tasks such as splicing site and alternative polyadenylation site prediction, and (2) a newly constructed RNA-seq splicing site dataset for *Arabidopsis thaliana*. The general workflow of these experiments is described in Figure 1.

### Overlapping vs. non-overlapping *k*-mer tokenization strategy

In our study, we used three different *k*-mer tokenization strategies: non-overlapping *k*-mers, fully overlapping *k*-mers, and 6-mer tokenization proposed by AgroNT (Mendoza-Revilla et al 2024). Each method tokenizes genomic sequences composed of the four nucleotides *A*, *T*, *C*, and *G* (Andrzej and Marek 2007) into small tokens containing *k* nucleotides, where *k* ranges from three to eight. This tokenization is more efficient than one-hot encoding and dna2vec (Ng 2017; Zhang et al 2023b).

**Fig. 2:**
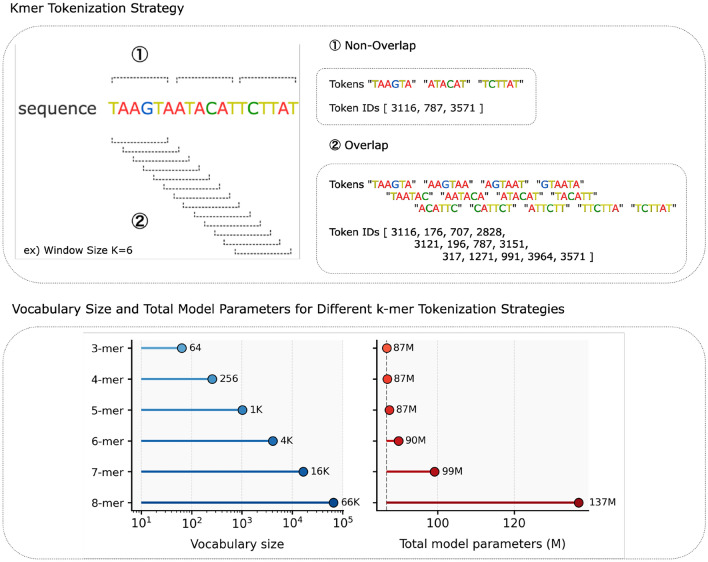
Illustration of overlapping and non-overlapping *k*-mer tokenization strategies

More specifically, in the case of fully overlapping, a token of length *k* is extracted by sliding the window by one nucleotide, while non-overlapping *k*-mers do not share nucleotides; e.g., from “ATGCCT”, “ATG,” “TGC,” “GCC,” and “CCT,” are extracted in the case of fully-overlapping, while “ATG” and “CCT” are extracted in the case of non-overlapping when $k = 3$.

AgroNT utilizes a non-overlapping 6-mer tokenization strategy. This method splits the genomic sequence into non-overlapping 6-mer tokens when possible. If 6-mer tokens cannot be generated (for example, when encountering *N* bases or at the end of the sequence), the sequence is split into single nucleotides. This approach minimizes token redundancy and reduces the total number of tokens but can potentially lose context when sequences are fragmented.

The vocabulary size $V_k$ for *k*-mer tokenization, including five special tokens, is then calculated by:

$$\begin{aligned} V_k = 4^k + 5 \end{aligned}$$

where $4^k$ represents all possible *k*-mers composed of the four nucleotides, and 5 represents the special tokens ([PAD], [MASK], [CLS], [SEP], and [UNK]).

For a genomic sequence of length *L*, the number of tokens $T_k$ generated by each tokenization method can be expressed as follows: 

*Non-overlapping k-mers:*

$$\begin{aligned} T_k = \lceil \frac{L}{k}\rceil + 2 \end{aligned}$$

*Fully-overlapping k-mers:*

$$\begin{aligned} T_k = L - k + 1 + 2 \end{aligned}$$

For AgroNT’s method, the number of tokens usually follows the non-overlapping 6-mer methods, but depends on the location of *N*s. In these equations, we add two to account for the [CLS] and [SEP] tokens that are typically used at the start and end of sequences in transformer models.

The choice of *k* significantly affects both the size of the vocabulary and the number of tokens generated (see Figure 2). For example, increasing *k* from three to eight expands the vocabulary size from 69 to 65541. This increase in *k* reduces the number of tokens generated for non-overlapping *k*-mers and increases it for fully overlapping *k*-mers, and it shows the trade-offs inherent in different tokenization strategies. In this study, we use 3- to 8-mer to capture more efficient genomic representations and optimize *k* windows.

### Pretraining Strategy and Model Architecture

We pre-train BERT (Devlin et al 2019) models implemented using Hugging Face Transformers (Wolf et al 2020) with various *k*-mer tokenizers, carefully selecting pre-training data distributions aligned with the downstream tasks. Given the substantial computational resources required for full-scale pre-training, we adopt a lightweight pre-training strategy by utilizing a small in-domain corpus (Sanchez and Zhang 2022) derived from plant-specific reference genomes.

**Fig. 3:**
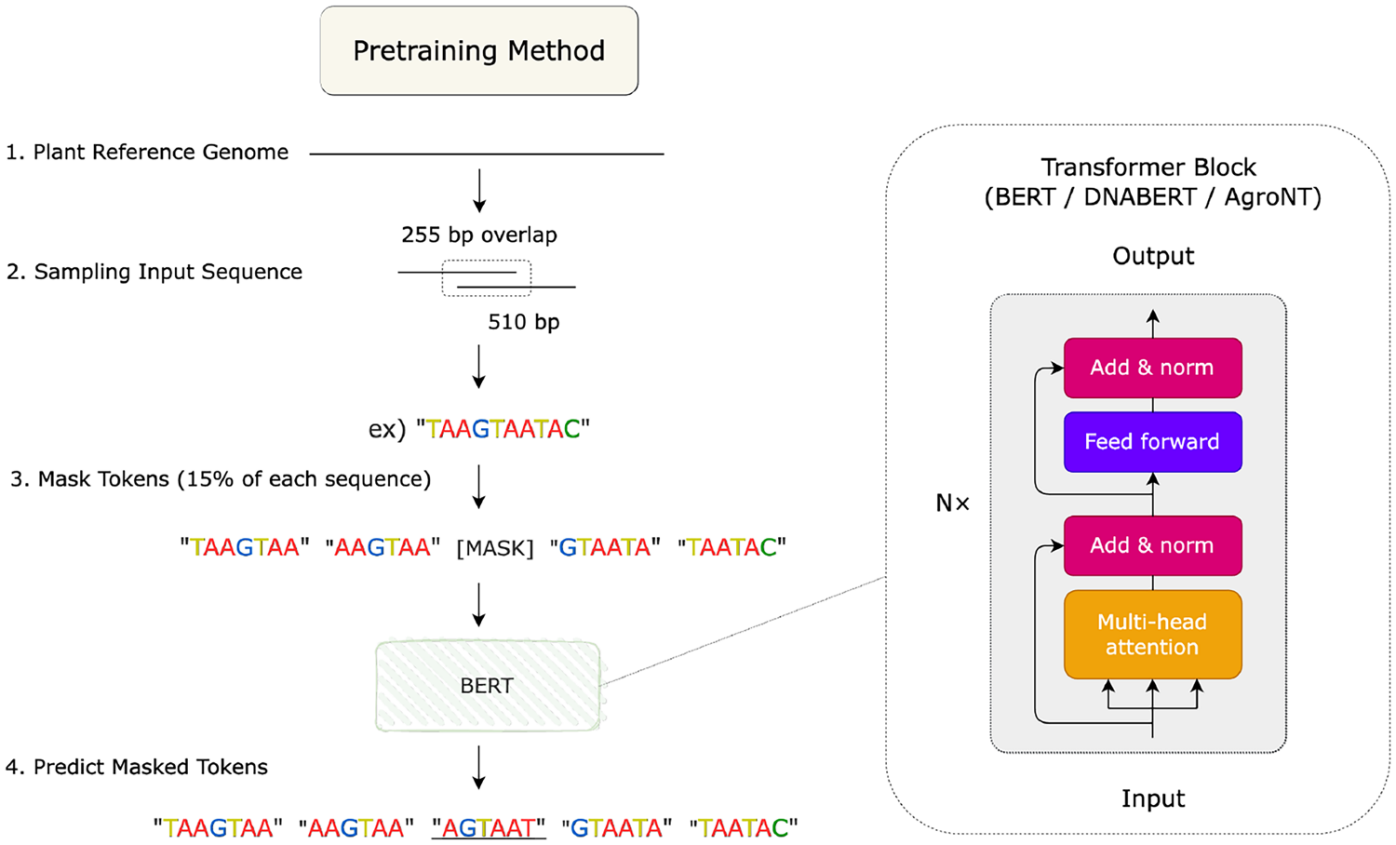
Overview of the pretraining strategy and model architecture

The pretraining corpus comprises reference genomes from six representative plant species: *Arabidopsis thaliana*, *Solanum lycopersicum*, *Oryza sativa*, *Glycine max*, *Sorghum bicolor*, and *Zea mays*. These species, selected from the NCBI RefSeq database (O’Leary et al 2016), include both model organisms and major crops, and were chosen to minimally cover the diversity of evolutionary distances and genomic characteristics within the plant kingdom.

As illustrated in Figure 3, we pre-trained BERT models using six different *k*-mer window sizes (*k*=3–8), each with fully overlapping tokenization. During task-specific fine-tuning, we evaluated both *overlapping* and *non-overlapping* variants derived from each checkpoint, enabling us to assess how window size and tokenization scheme jointly affect downstream performance. During pretraining, we extracted 510 bp subsequences from the reference genomes with a stride of 255 bp, ensuring 50% overlap between adjacent subsequences. Each subsequence was tokenized using the respective *k*-mer window size with full overlap.

We applied a masking rate of 15% during pretraining to train the models in a self-supervised fashion. Specifically, random *k*-mers were masked and the model was trained to predict the original tokens, encouraging the model to learn contextual dependencies in the sequence. Each model was trained for 50–80*k* steps, or until convergence of the loss function.

### Evaluation dataset and metrics

We evaluated all *k*-mer tokenizer models using a diverse set of plant genomic benchmarks (Mendoza-Revilla et al 2024), along with an additional RNA-seq-based dataset curated in this study (Section 2.3.2). These datasets span several plant species, including *Arabidopsis thaliana*, *Oryza sativa*, *Trifolium pratense*, *Medicago truncatula*, and *Chlamydomonas reinhardtii*, and cover a range of tasks such as splice site prediction, alternative polyadenylation site prediction, promoter strength prediction, and terminator strength prediction, as summarized in Table 1.

**Table 1:** Details of the evaluation datasets used in this study

Task name	Species and details	Seq. (bp)	Data source	Task type	Metrics
Splicing	*A. thaliana* (donor)	398	Baten et al (2006)	Bin. Class	MCC
	*A. thaliana* (acceptor)				
Splicing (RNA-seq)	*A. thaliana*	160	This study	3-Class	Macro-averaged
	(donor & acceptor)				PR and ROC
Polyadenylation	*A. thaliana*	400	Mendoza-Revilla et al (2024)	Bin. Class	MCC
	*O. sativa* (indica group)				
	*T. pratense*				
	*M. truncatula*				
	*C. reinhardtii*				
	*O. sativa* (japonica group)				
Promoter Strength	Tobacco Leaf	170	Jores et al (2021)	Regression	$R^2$
	Maize Protoplast				
Terminator Strength	Tobacco Leaf	170	Gorjifard et al (2024)	Regression	$R^2$
	Maize Protoplast				

Bin. Class.: Binary Classification, 3-Class.: 3-Class Classification, *PR* Precision-Recall, *ROC* Receiver operating characteristic, *MCC* Matthews correlation coefficient, $R^2$: Coefficient of Determination

#### Plant genomic benchmark task details

While the plant genomic benchmark covers a broad range of tasks, we selected four that are applicable to all *k*-mer tokenizer models, in order to avoid limitations imposed by model-specific token length constraints. For splicing and polyadenylation tasks, where validation splits were not available, we used 10% of the training data as a validation set.

*Splice Site Prediction* This task focuses on identifying splice sites in the *A. thaliana* genome. The dataset was compiled by Baten et al. (Baten et al 2006), consists of 398 bp sequences containing both acceptor and donor sites, as well as non-acceptor and non-donor sequences. The dataset is highly imbalanced, with significantly more non-splice site sequences than splice site sequences.

*Alternative Polyadenylation Site Prediction* This task involves predicting alternative polyadenylation (APA) sites in plant genomes. The dataset, obtained from PlantAPAdb (Zhu et al 2020), includes sequences from five plant species and reconstructed (Gao et al 2018; Loke et al 2005): *O. sativa*. (japonica and indica), *A. thaliana*, *C. reinhardtii*, *M. truncatula*, and *T. pratense*. Each sequence is 400 bp long, with the polyadenylation site at the 301st position. Negative samples were generated by shifting the position of the polyadenylation site.

*Promoter Strength Prediction* This task involves predicting the strength of promoter regions in plant genomes. The dataset is based on self-transcribing active regulatory region sequencing (STARR-seq) assays (Muerdter et al 2015) for *A. thaliana*, *Z. mays*, and *S. bicolor* (Jores et al 2021). It includes 170 bp promoter sequences, defined as -165 to +5 bp relative to annotated transcription start sites (TSS), and their measured strength in two systems: tobacco leaves and maize protoplasts.

*Terminator Strength Prediction* This task aims to predict the activity of the terminator regions. Data come from a study based on STARR-seq that measured the activity of more than 50,000 terminators from *A. thaliana* and *Z. mays* (Gorjifard et al 2024). These terminators were defined as 170 nucleotide sequences spanning from position -150 to +20 relative to a cleavage and polyadenylation site.

#### RNA-seq splice site dataset construction pipeline

We constructed an updated *A. thaliana* splicing site prediction dataset from RNA-seq data, as the splicing task in the plant genomic benchmark source data dates back to 2006. RNA-seq data from following multiple tissues were integrated:

- *Shoot Apices:* wild-type shoot apex tissue from (Lee et al 2023), studying the role of splicing factors AtU2AF65a and AtU2AF65b in flowering time regulation.
- *Rosette Leaves:* wild-type rosette leaves from (Scherer et al 2024), analyzing the role of the cytochrome b6f complex in photosynthesis.
- *Primary Roots:* wild-type primary root tissue from (Cavalleri et al 2024), investigating RNA-directed DNA methylation pathways mediated by the MOM1 complex.
- *Flower Buds:* wild-type flower buds from (Li et al 2023), examining the impact of uracil phosphoribosyltransferase on chloroplast function during floral development.

The dataset construction proceeded as follows. Raw SRA files were downloaded from NCBI using sratools and converted to FASTQ format via fasterq-dump. Quality control–including adapter trimming and removal of low-quality bases–was performed with fastp (Chen et al 2018). High-quality reads were then aligned to the *A. thaliana* reference genome using STAR (Dobin et al 2013) with a custom-built index and splice-aware parameters. Splice junctions were extracted from the alignment outputs, and junctions with coordinate differences within $\pm 2$ bp were merged to consolidate similar events using bedtools (Quinlan and Hall 2010).

For each merged splice junction, a 160 bp sequence window (80 bp upstream and 80 bp downstream of the junction) was extracted from the reference genome to serve as a positive example. Negative samples were sampled by randomly selecting genomic regions that do not overlap any annotated splice junctions. The final dataset comprises sequences centered on donor sites (5’ splice sites), acceptor sites (3’ splice sites), and negative examples.

To facilitate robust model training and evaluation, the dataset was partitioned by chromosome: chromosomes 1–3 form the training set, chromosome 4 is reserved for validation, and chromosome 5 (and any additional chromosomes) constitutes the test set.

### Fine-tuning method and hyperparameters

We performed full fine-tuning for the BERT pre-trained on plant genomes and DNABERT models, updating all parameters as is standard practice. For the AgroNT model, achieving the originally reported performance required careful adaptation due to the unavailability of its specific fine-tuning code and significant differences in our computational environment. To ensure the best possible reproduction of its performance under our settings, we applied full fine-tuning where feasible and additionally employed two parameter-efficient fine-tuning methods, LoRA (Hu et al 2022) and $\mathrm {(IA)}^3$ (Liu et al 2022). Furthermore, we accounted for differences from the original experimental setup, particularly regarding validation data. For the splicing and polyadenylation tasks, we used 10% of the training data as a validation set, a setup which differs from that described in the original AgroNT paper for certain tasks. We confirmed the reproducibility under these modified conditions through preliminary experiments, as detailed in Appendix Table3 and 4 in Appendix 7.

The adopted hyperparameters are summarized as follows. For full fine-tuning, following the respective original papers, we used learning rates of $2 \times 10^{-5}$ for BERT and $3 \times 10^{-5}$ for DNABERT. The number of training epochs was set to 3 for the splicing task, 5 for the polyadenylation task, and 10 for both the promoter and terminator strength prediction tasks. For LoRA, we used a learning rate of $2 \times 10^{-4}$, rank $r=8$, scaling factor $\alpha =16$, and trained for 4 epochs. LoRA adapters were applied to the query ($W_q$), key ($W_k$), value ($W_v$), and output ($W_o$) projection matrices. For $\mathrm {(IA)}^3$, the learning rate was $3 \times 10^{-3}$ with 4 training epochs, and the adapters were applied to $W_q$, $W_v$, and $W_o$. All experiments utilized the AdamW optimizer, and training was performed using mixed-precision.

For the RNA-seq splicing dataset specifically, we employed a batch size of 128. The learning rate was set to $3 \times 10^{-3}$ for full fine-tuning, and $1 \times 10^{-2}$ for both LoRA and $\mathrm {(IA)}^3$. Training proceeded for a maximum of 300 steps using the scheduler-free RAdam optimizer (Defazio et al 2024). Computations for this dataset were performed using bfloat16 precision. In this specific setting, LoRA was configured with rank $r=16$, $\alpha =32$, and applied to $W_q$, $W_k$, and $W_v$. The $\mathrm {(IA)}^3$ adapters were applied to $W_q$, $W_v$, and $W_o$.

## Results

### Splice and polyadenylation site benchmark results

**Fig. 4:**
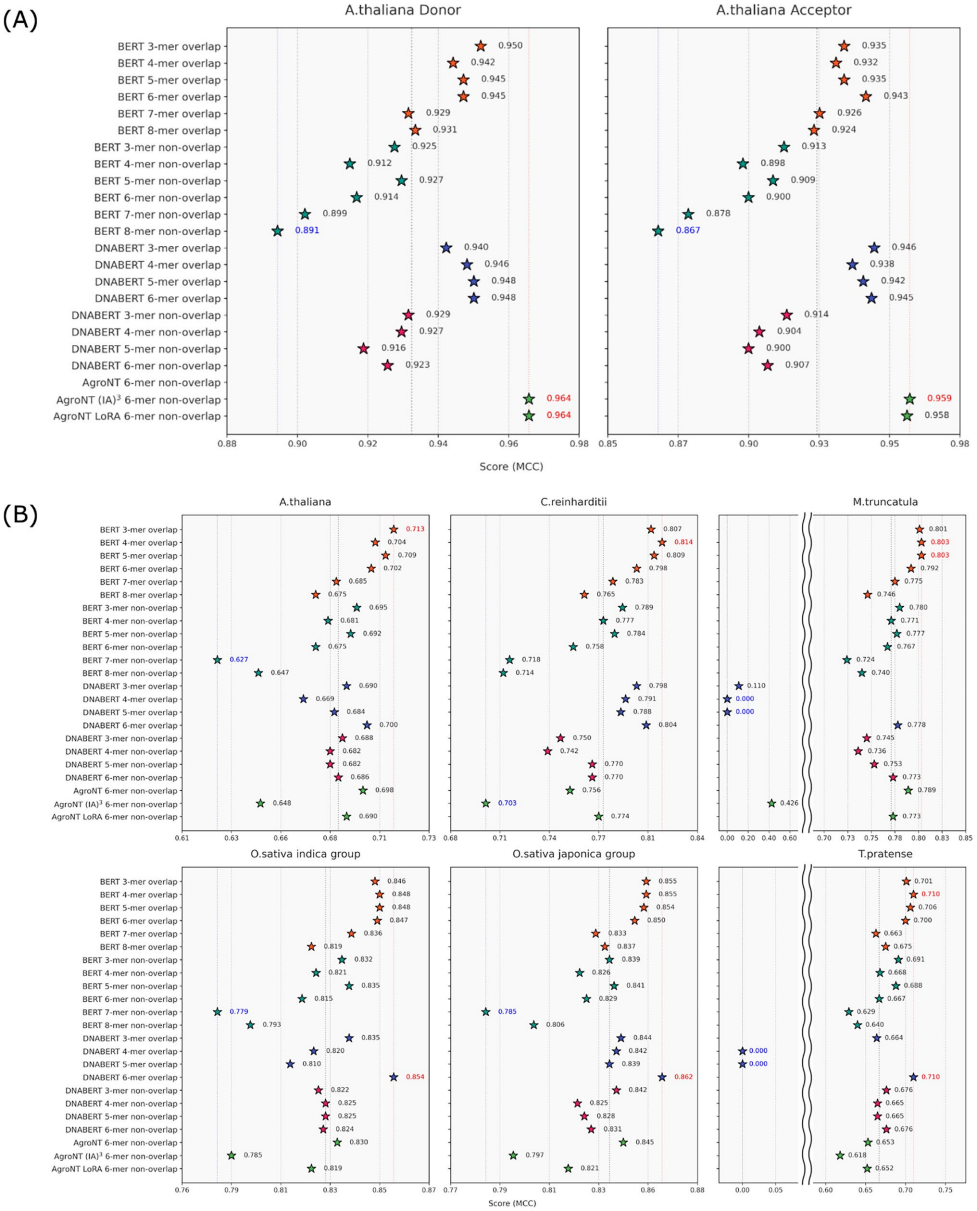
A. thaliana Splice and Polyadenylation Site Prediction Results: (**A**) Left: donor sites; right: acceptor sites. (**B**) Top row: *A. thaliana*, *C. reinhardtii*, *M. truncatula*. Bottom row: *O. sativa* indica group, *O. sativa* japonica group, *T. pratense*

The benchmark results for splice site prediction and alternative polyadenylation site prediction are shown in Figures 4A and B. For splice site prediction, the BERT model pre-trained on plant genomes exhibited high performance when using an overlapping tokenizer (with *k*-mers ranging from 3 to 8). Specifically, the Matthews Correlation Coefficient (MCC) values ranged from 0.929 to 0.950 for donor sites and from 0.924 to 0.943 for acceptor sites. In contrast, using a non-overlapping tokenizer reduced the performance, with donor site MCC values between 0.891 and 0.927 and acceptor site MCC values between 0.867 and 0.913. Similarly, the DNABERT model pre-trained on the human genome (employing a 3- to 6-mer overlapping tokenizer) achieved comparable performance with MCC scores of 0.940$-$0.948 for donor sites and 0.938$-$0.946 for acceptor sites. Notably, the AgroNT model pre-trained on 48 edible plant species attained the highest accuracy overall, yielding donor site MCC values around 0.964 and acceptor site MCC values between 0.958 and 0.959 when fine-tuned using either the $\mathrm {(IA)}^3$ or LoRA approaches.

Regarding polyadenylation site prediction, the BERT model pre-trained on plant genomes using an overlapping *k*-mer tokenizer demonstrated consistent performance across different plant species. For instance, in *Arabidopsis thaliana*, a 3-mer overlap produced an MCC of 0.713; in *Chlamydomonas reinhardtii*, a 5-mer overlap resulted in an MCC of 0.814; in *Medicago truncatula*, both 4-mer and 5-mer overlaps yielded an MCC of 0.803; and in *Trifolium pratense*, a 4-mer overlap achieved an MCC of 0.710. While tokenizers with *k*-mers ranging from 3 to 6 generally provided similar accuracy, the use of 7-mer and 8-mer tokenizers tended to reduce performance. When a non-overlapping tokenizer was applied, overall prediction accuracy decreased further; although the performance for 3- to 6-mer tokenizers was nearly comparable to that of the overlapping approach, larger window sizes exacerbated the decline in accuracy. Conversely, the DNABERT model demonstrated its best performance on *Oryza sativa* (both indica and japonica groups) with a 6-mer overlapping tokenizer, achieving MCC values of 0.854 and 0.862, respectively. However, for *M. truncatula*, overlapping tokenizers with 3- to 5-mer settings resulted in MCC scores as low as 0.000$-$0.110, and in *T. pratense*, 4-mer and 5-mer overlaps led to an MCC of 0.000, indicating a complete prediction failure in those cases. The AgroNT model, when fine-tuned using either conventional methods or LoRA, maintained stable accuracy across all species; however, fine-tuning with $\mathrm {(IA)}^3$ resulted in an overall performance drop.

### Promoter and terminator strength benchmark results

**Fig. 5:**
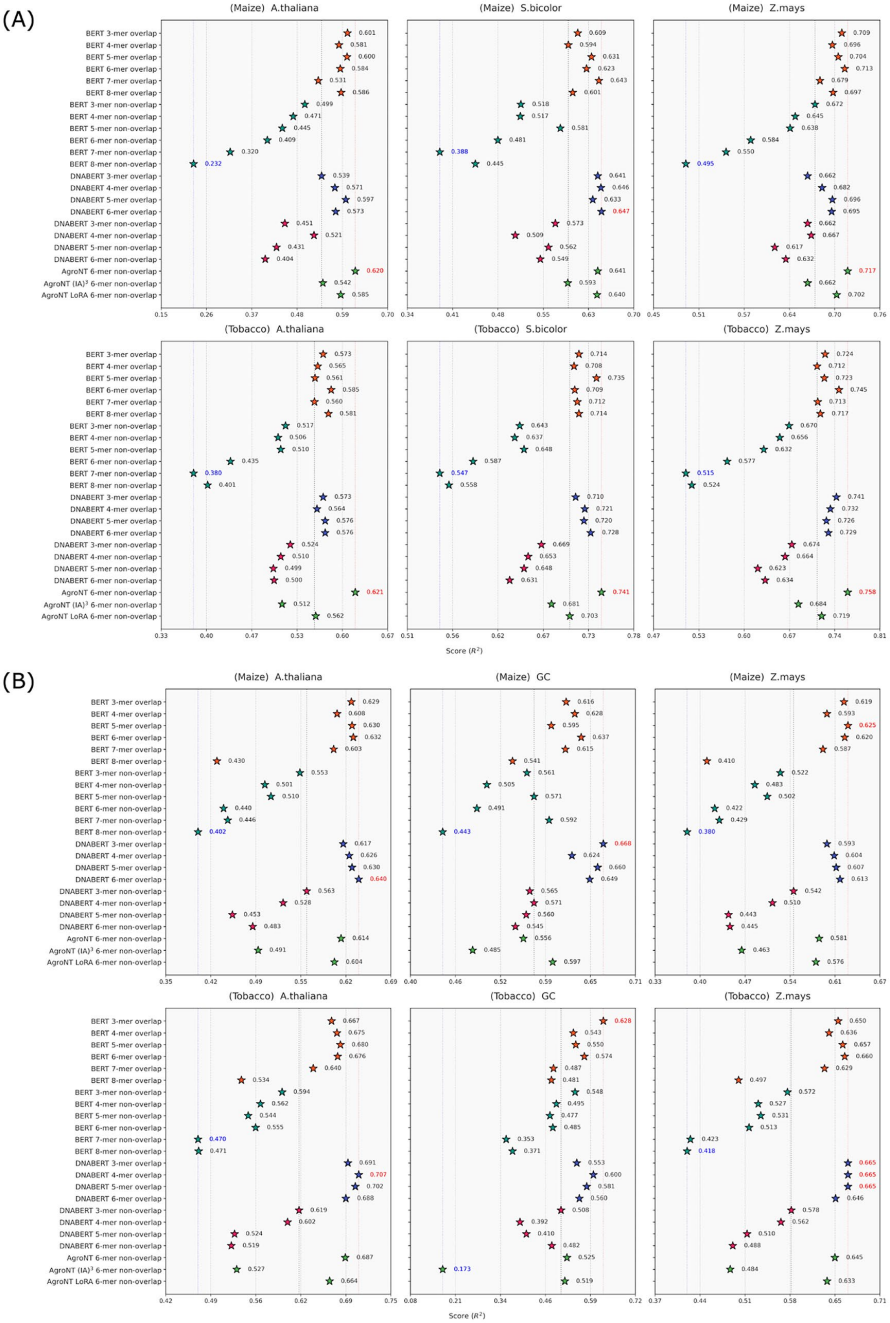
Promoter and Terminator Strength Prediction Results: (**A**) Top: maize model; bottom: tobacco leaf system. Columns represent *A. thaliana*, *S. bicolor*, and *Z. mays*. (**B**) Top: maize model; bottom: tobacco leaf system. Columns represent *A. thaliana*, GC-controlled sequences (GC), and *Z. mays*

The benchmark results for promoter strength and terminator strength prediction tasks are presented in Figures 5A and B. For promoter strength prediction, the BERT model pre-trained on plant genomes consistently achieved high prediction accuracy, especially when an overlapping *k*-mer tokenizer was used. Comparable performance was observed across window sizes from 3 to 8; however, employing a non-overlapping tokenizer led to inferior accuracy that tended to decline as the window size increased–a trend also seen in the human genome-pretrained DNABERT model. For example, in the maize protoplast system, the promoter from *Sorghum bicolor* reached the highest $R^2$ value of 0.647 with a 6-mer overlapping tokenizer. Overall, the prediction accuracy did not differ significantly between the plant-derived and human-derived BERT models. In contrast, the AgroNT model–despite using a 6-mer non-overlapping tokenizer–attained the highest prediction accuracy across all systems when fine-tuned using conventional methods. Specifically, in the maize protoplast system, promoters from *A. thaliana* and *Zea mays* achieved $R^2$ values of 0.620 and 0.717, respectively, while in the tobacco leaf system, promoters from *A. thaliana*, *S. bicolor*, and *Z. mays* reached $R^2$ values of 0.621, 0.741, and 0.758, respectively. On average, AgroNT outperformed conventional CNN-based models by approximately three $R^2$ points.

For terminator strength prediction, the BERT model pre-trained on plant genomes also demonstrated consistently high accuracy when using overlapping tokenizers with *k*-mer sizes ranging from 3 to 6. For instance, in the maize protoplast system, the terminator from *Z. mays* obtained an $R^2$ of 0.625 with a 5-mer overlapping tokenizer, whereas in the tobacco leaf system, the terminator (accounting for GC content) achieved an $R^2$ of 0.628 with a 3-mer overlapping tokenizer; these were the highest scores observed across all models. However, as with the other tasks, using 7-mer and 8-mer tokenizers reduced accuracy, and non-overlapping tokenizers further exacerbated the decline with increased window sizes. The DNABERT model exhibited similar behavior; in the maize protoplast system, the *A. thaliana* terminator reached an $R^2$ of 0.640 with a 6-mer overlapping tokenizer, while the GC-controlled terminator achieved an $R^2$ of 0.668 with a 3-mer overlapping tokenizer. Furthermore, in the tobacco leaf system, the *A. thaliana* terminator achieved an $R^2$ of 0.707 with a 4-mer overlapping tokenizer, and the *Z. mays* terminator reached an $R^2$ of 0.665 with overlapping tokenizers of sizes 3 to 5–the highest scores among all models. The AgroNT model, using a 6-mer non-overlapping tokenizer, reached prediction accuracies comparable to those of the BERT and DNABERT models (which used overlapping tokenizers) when fine-tuned using either conventional methods or LoRA.

### Updated Splice Site Prediction Results Using RNA-seq Data

**Fig. 6:**
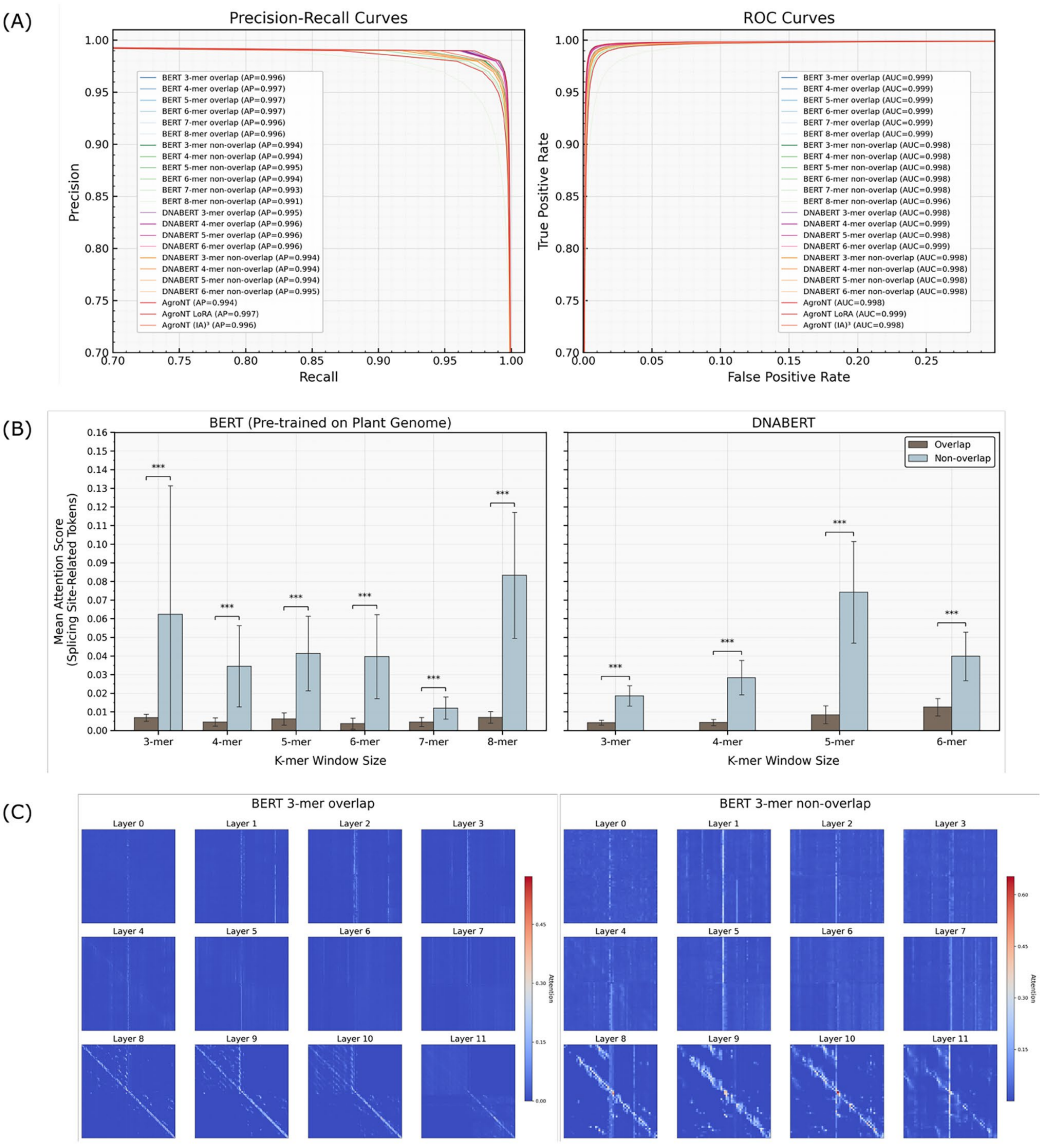
Prediction Results for *A. thaliana* Splice Site Detection: (**A**) Multi-class precision-recall curves (left) and receiver operating characteristic curves (right). (**B**) Mean attention scores for splicing site-associated tokens (***: p-value < 0.001), comparing the BERT model pre-trained on a plant genome corpus (left) with DNABERT pre-trained on a human genome corpus (right). (C) Example attention maps from the BERT model pre-trained on plant genomes 3-mer model, illustrating overlapping (left) versus non-overlapping (right) tokenization across all layers. The input sequence corresponds to the first donor site in the test set

Figure 6A presents the results of splice site prediction. For the BERT model pre-trained on plant genomes, the macro-average precision (MAP) achieved with overlapping tokenization ranged from 0.996 to 0.997 across 3-mer to 8-mer configurations. In contrast, the non-overlapping tokenizer yielded slightly lower MAP values, ranging from 0.991 to 0.995. Similarly, DNABERT achieved MAP values of 0.995-$-$0.996 with overlapping tokenization (3-mer to 6-mer), and 0.994-$-$0.995 with non-overlapping tokenization. For the AgroNT model, standard fine-tuning resulted in a MAP of 0.994, while LoRA achieved 0.997 and $\mathrm {(IA)}^3$ reached 0.996. In all cases, macro-average ROC values were consistently high, ranging between 0.998 and 0.999.

Next, we extracted, for each test sample and each *k*-mer window size, the mean attention score at the final layer for tokens overlapping true splice sites. We then compared these scores between overlapping and non-overlapping tokenizers using Welch’s two-sided t-test (unequal variances) for each model-*k* combination (Figure 6B). All raw p-values were adjusted via the Benjamini-Hochberg procedure to control the false discovery rate across comparisons. In both the plant-pretrained BERT and DNABERT models, non-overlapping tokenizers yielded consistently higher attention scores, and the aggregated FDR-corrected p-value was below 0.001, demonstrating a statistically significant advantage in attention localization for the non-overlapping strategy.

We further visualized the attention maps across all layers for representative input sequences containing a 5$'$ splice site, comparing overlapping and non-overlapping tokenization using the 3-mer BERT model (Figure 6C). The overlapping tokenizer exhibited lower attention to splice sites and a more uniform distribution of attention scores across tokens, likely due to the high redundancy among adjacent tokens and the increased number of tokens. In contrast, the non-overlapping tokenizer yielded markedly higher attention scores at splice sites, particularly in the final layer.

**Fig. 7:**
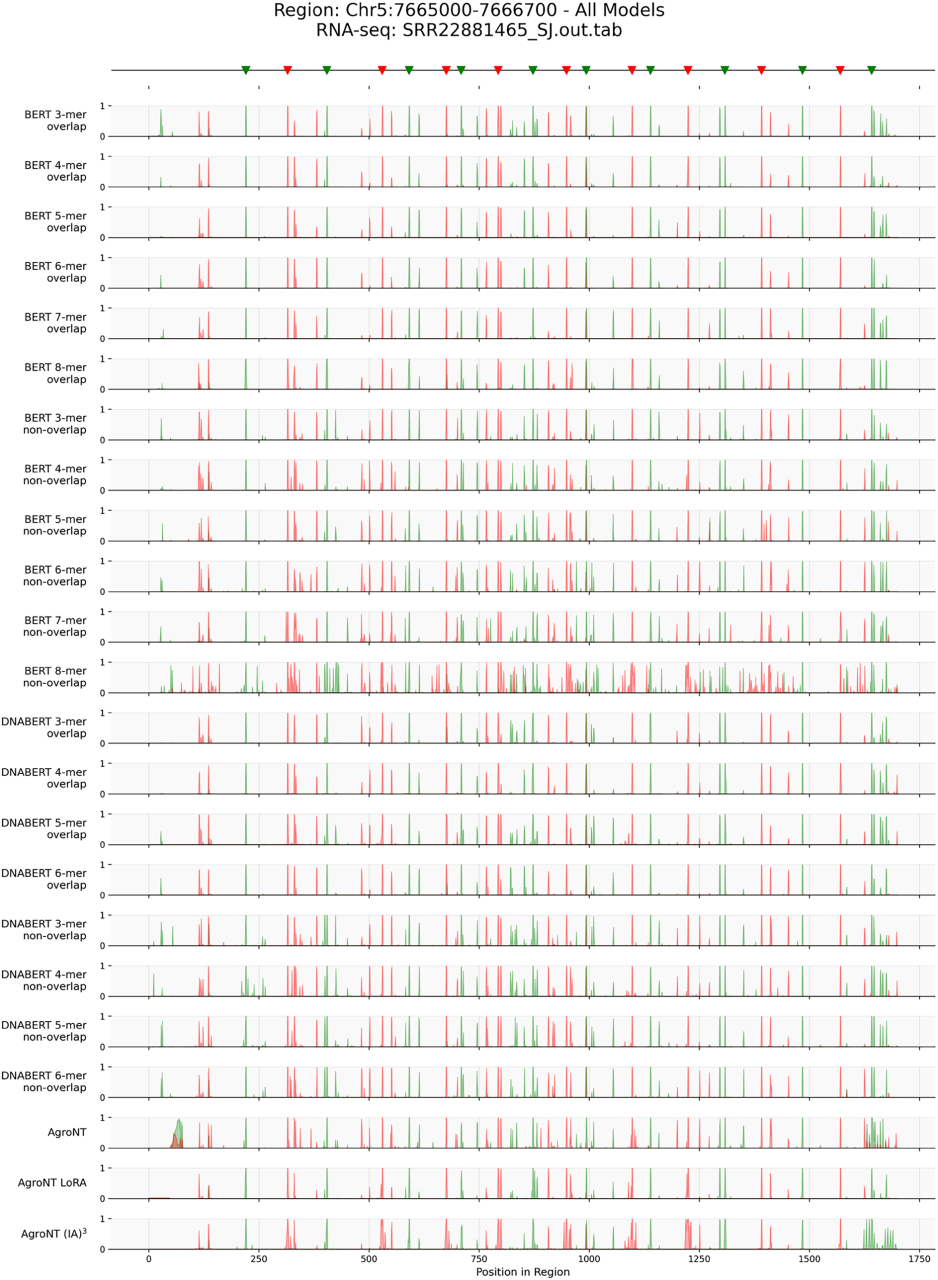
Example Genome Annotation Inference Results Across All *k*-mer Tokenization Models: The top panel displays RNA-seq splice junction annotations from SRR22881465 (chr5: 7665000–7666700), with donor sites shown in green and acceptor sites in red. The bottom panels present the predicted logits from each model, where green indicates donor site predictions and red indicates acceptor site predictions

Finally, genome annotation inference was conducted on a test region (SRR22881465 RNA-seq, chromosome 5: 7665000–7666700) using all models and tokenizers (Figure 7). All models successfully identified the true 5$'$ and 3$'$ splice sites. However, non-overlapping tokenizers showed increased false positives, especially with larger *k*-mer sizes. This trend was most pronounced in the plant-pretrained BERT model. In contrast, overlapping tokenizers showed stable prediction logits across all window sizes. Among all models, the AgroNT model fine-tuned with LoRA—which showed the highest prediction accuracy in Figure 6A—exhibited the lowest false positive rate and most precise inference.

It is also worth noting that all models were trained on datasets where the number of negative samples was approximately twice the number of positive splice sites (5$'$ and 3$'$ combined). We expect that increasing the proportion of negative samples to better reflect real genome annotation distributions would reduce false positive predictions in practice.

## Discussion

In this study, we evaluated the performance of various transformer-based genomic language models (gLMs) employing different *k*-mer tokenization strategies for genomic sequence analysis across multiple plant species. Our findings suggest that overlapping *k*-mer tokenization consistently enhances performance in tasks that require capturing local sequence context, such as splice site detection and promoter or terminator strength prediction in Figures 4 and 5. This advantage is likely due to the preservation of critical local motifs within genomic sequences.

*Relationship Between Genomic Features and Tokenization Strategies* The effectiveness of overlapping tokenization can be attributed to the intrinsic features of various genomic elements:

- *Splice Sites:* Defined by short sequence motifs (typically 6–10 nucleotides) at exon-intron boundaries, splice sites rely on precise base-pairing interactions with snRNAs and the binding of splicing factors (Sheth et al 2006).
- *Alternative Polyadenylation Sites:* The canonical polyadenylation signal (“AAUAAA”) and its downstream GU-rich region span a broader area, making accurate detection more challenging (Stroup and Ji 2023; Liz and Hunt 1997).
- *Promoters:* Core promoter elements such as the TATA-box, initiator, and downstream promoter element occur within defined regions relative to the transcription start site (Jores et al 2021).
- *Terminators:* Terminator strength is influenced by sequence features surrounding the cleavage and polyadenylation site, including upstream U-rich or A-rich elements and downstream GU- or U-rich regions (Gorjifard et al 2024).

These observations indicate that tokenization strategies should be carefully chosen based on the genomic context of the task. Nevertheless, the use of overlapping tokenizers may result in highly similar adjacent tokens, which could lead to redundant information representation in certain tasks or model configurations (Figure 4B: M. truncatula, T. pratense).

*Attention Mechanisms and Trade-offs* Analysis of attention scores in the RNA-seq-derived splicing dataset revealed that non-overlapping tokenization yielded higher attention scores around true splice sites (Figure 6B), particularly in deeper transformer layers (Figure 6C). This suggests that aligning token boundaries with known sequence motifs improves the localization of critical features. However, the non-overlapping approach also resulted in increased false positive predictions during genome annotation inference (Figure 7), highlighting a trade-off between enhanced interpretability and prediction robustness.

*Optimal* *k-mer Size and Computational Considerations* Interestingly, the optimal *k*-mer window size for our gLMs differed from traditional *k*-mer analyses, where larger *k* values generally lead to improved accuracy (Moeckel et al 2024; Shaw and Yu 2022). In our experiments, smaller *k*-mers provided better sequence coverage with non-overlapping tokenization, whereas larger *k*-mers offered computational efficiency benefits. These findings underscore the need to balance sequence coverage and computational demands when selecting the optimal *k*-mer size and overlap strategy for specific research objectives.

*Limitations and Future Directions* Despite the strong performance achieved by the BERT-based models with overlapping tokenization, several limitations remain. The quadratic computational complexity (Keles et al 2023) and input length constraints of transformer architectures restrict their applicability to very long sequences (for example, long non-coding RNA detection or genome-wide species classification), thereby increasing resource demands. Furthermore, while our optimized smaller models show promise, they may not fully replicate the capabilities of large-scale models such as AgroNT, particularly in scenarios like zero-shot learning (Mendoza-Revilla et al 2024). Additionally, challenges in reproducing AgroNT’s performance and optimizing parameter-efficient fine-tuning methods call for further investigation.

Future work should explore optimizing pretraining strategies. Although pretraining on domain-specific data–as performed in this study–can better align data distributions (Gururangan et al 2020), the overall necessity of extensive pretraining for genomic language models remains debatable (Vishniakov et al 2025). Direct comparisons between domain-specific pretraining and fine-tuning of existing models will be crucial.

Moreover, improving model efficiency and input flexibility using methods such as attention with linear biases (Press et al 2022), flash-attention (Dao et al 2022), and local window attention is important. Future studies should also investigate knowledge transfer and systematically compare representations derived from different tokenization methods (e.g., single nucleotide, *k*-mer, and byte-pair encoding). Finally, visualizing the relationship between genomic features and tokenization effects may provide valuable insights for designing more efficient and accurate genomic language models.

## Conclusion

We demonstrate that transformer-based gLMs with carefully designed *k*-mer tokenization strategies can achieve performance comparable to that of much larger models across a range of plant genomic prediction tasks. This finding is significant for the plant science community, as it offers advanced genomic analysis capabilities with models that are computationally more accessible to researchers operating under resource constraints.

It is important to note that while our experiments did not address zero-shot prediction, previous work has shown that large-scale models such as AgroNT exhibit remarkable versatility across broader tasks, including zero-shot gene expression prediction. In other words, although the overlapping *k*-mer tokenization models achieve high performance in the evaluated tasks, models like AgroNT bring the advantage of generalizability and broader applicability for diverse genomic challenges.

Our study contributes to the ongoing efforts to optimize machine learning approaches in plant genome analysis. Looking ahead, large-scale gLMs remain a powerful option; however, simply scaling up model parameters is not sufficient to address the multifaceted challenges inherent in genomics. To uncover novel genetic events and elucidate regulatory mechanisms, future research should explore more effective problem formulations, refined model architectures, and innovative tokenization strategies. By integrating the strengths of computationally efficient, task-focused models with the versatility demonstrated by larger models, the field can achieve improved accuracy and more explanatory visualizations *in silico* analyses–ultimately accelerating progress in plant biology.

## Figures and Tables

**Fig. 8:**
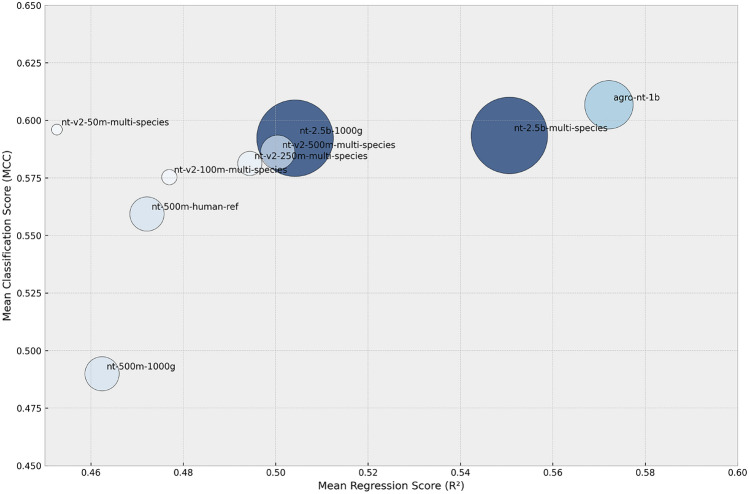
The Nucleotide Transformer model results on plant genomic benchmarks. Each circle represents the number of model parameters

## Data Availability

The plant genomic benchmark datasets are available on the Hugging Face Hub: InstaDeepAI/plant-genomic-benchmark. The splicing dataset constructed from *A. thaliana* RNA-seq data can be accessed at suzuki-2001/athaliana_rnaseq_splicing. Pre-trained BERT models are provided in the Hugging Face collection: suzuki-2001/kmer-tokenization-strategy. The source code for k-mer tokenization, fine-tuning, and dataset construction is available at: suzuki-2001/pmb2025-kmer-tokenization-strategy.

## A Model Plant Reference Genome Corpus

Appendix Table 2 summarizes the plant species included in our pre-training corpus, along with their reference genome versions and genome sizes. This selection represents a diverse set of plant genomes that were incorporated into the model to enhance its domain-specific understanding.

**Table 2:** Details of the plant genome corpus used for model pre-training

Species	Reference Version	Genome Size
*A. thaliana*	TAIR10.1	119 Mb
*S. lycopersicum*	SL3.0	828 Mb
*O. sativa*	IRGSP$-$1.0	373 Mb
*G. max*	v4.0	978 Mb
*S. bicolor*	NCBIv3	708 Mb
*Z. mays*	NAM$-$5.0	2.2 Gb

## B Preliminary Experiments of Agronomic Nucleotide Transformer Performance

For the splicing and polyadenylation tasks in the plant genomic benchmark, we used 10% of the training data as a validation set. Although this approach may limit direct comparisons with other methods, we endeavored to replicate the performance reported for AgroNT under similar conditions. Appendix Tables 3 and 4 summarize our results, which are comparable to the original baseline. Nevertheless, these outcomes should be interpreted with caution due to potential discrepancies in data splitting.

**Table 3:** Preliminary results on splice site prediction

Method	*A. thaliana* donor	*A. thaliana* acceptor
Reported	0.95 / 0.98	0.97 / 0.97
FT	OOM	OOM
LoRA	0.99 / 0.99	0.99 / 0.98
$(\textrm{IA})^3$	0.99 / 0.99	0.99 / 0.98

*Values are reported as AUC / AUPRC. OOM: Out of Memory

**Table 4:** Preliminary results on alternative polyadenylation site prediction

Method	*A. thaliana*	*O. sativa* (I)	*T. pratense*	*M. truncatula*	*C. reinhardtii*	*O. sativa* (J)
Reported	0.92 / 0.87	0.96 / 0.92	0.89 / 0.82	0.95 / 0.90	0.94 / 0.89	0.96 / 0.93
FT	0.84 / 0.84	0.91 / 0.91	0.81 / 0.81	0.90 / 0.88	0.88 / 0.86	0.92 / 0.91
LoRA	0.92 / 0.87	0.97 / 0.94	0.82 / 0.81	0.88 / 0.88	0.88 / 0.88	0.91 / 0.90
$(\textrm{IA})^3$	0.90 / 0.84	0.96 / 0.92	0.89 / 0.82	0.81 / 0.66	0.93 / 0.88	0.96 / 0.93

*Values are reported as AUC / AUPRC (I): Indica group, (J): Japonica group

## C Benchmarking DNABERT Variants on Plant Genomic Tasks

DNABERT has three main variants: DNABERT (Ji et al 2021), DNABERT-2 (Zhou et al 2023), and DNABERT-S (Zhou et al 2024). The DNABERT-S variant extends DNABERT-2 with species-aware training strategies ($\hbox {C}^2$LR). While DNABERT-2 and DNABERT-S employ byte-pair encoding (BPE) tokenization and have shown strong performance on human genomic tasks and on updated splice site prediction, their application to certain plant genomic benchmark tasks such as splice site prediction and alternative polyadenylation site prediction has proven more challenging. The following factors may help contextualize the observed performance discrepancies.

1. *Variable Length Tokenization*: The BPE tokenizer splits sequences into variable length subwords. Although this flexibility can be advantageous, it may misalign short motifs with token boundaries, making precise motif detection harder than with fixed length *k*-mers.
2. *Relative Positional Bias (ALiBi)*: Replacing absolute positional embeddings with ALiBi’s relative biases means that the model no longer encodes exact base indices (e.g. the 40^th^ base), potentially reducing spatial resolution for tasks that depend on fixed positions. This may particularly affect polyadenylation site prediction, where canonical motifs and downstream elements occur at defined distances from cleavage sites. Consequently, the model may struggle to localize these signals with high precision.

In summary, the choices of BPE tokenization and ALiBi positional encoding in DNABERT-2 and DNABERT-S may not be optimal for certain plant genomic tasks, especially those requiring highly precise motif localization such as polyadenylation site prediction. These considerations remain hypotheses for further investigation rather than definitive conclusions (Tables 5, 6, 7, 8, 9, 10).

**Table 5:** A. thaliana Splice Site Prediction (MCC)

Method	donor (PGB)	acceptor (PGB)	donor/acceptor (RNA-seq)	*Human donor	*Human acceptor
DNABERT-2	0.0	0.0	0.926	0.836	0.831
DNABERT-S	0.0	0.0	0.923	0.841	0.835

PGB: Plant Genomic Benchmark

*Nucleotide Transformer downstream tasks (revised version): Human annotated splice sites from GENCODE V44 gene annotation. We used 10% of the training data for validation

**Table 6:** Alternative Polyadenylation Site Prediction (MCC)

Method	*A. thaliana*	*O. sativa (I)*	*T. pratense*	*M. truncatula*	*C. reinhardtii*	*O. sativa (J)*
DNABERT-2	0.0	0.0	0.0	0.0	0.0	0.0
DNABERT-S	0.0	0.0	0.0	0.0	0.0	0.0

(I): Indica group, (J): Japonica group

**Table 7:** Promoter Strength Prediction (Maize Protoplast System, $\hbox {R}^2$)

Method	*A. thaliana*	*S. bicolor*	*Z. mays*	Avg.
DNABERT-2	0.551	0.618	0.693	0.621
DNABERT-S	0.540	0.622	0.697	0.620

**Table 8:** Promoter Strength Prediction (Tobacco Leaf System, $\hbox {R}^2$)

Method	*A. thaliana*	*S. bicolor*	*Z. mays*	Avg.
DNABERT-2	0.577	0.714	0.716	0.669
DNABERT-S	0.586	0.708	0.727	0.673

**Table 9:** Terminator Strength Prediction (Maize Protoplast System, $\hbox {R}^2$)

Method	*A. thaliana*	*S. bicolor*	*Z. mays*	Avg.
DNABERT-2	0.633	0.611	0.590	0.612
DNABERT-S	0.645	0.627	0.601	0.624

**Table 10:** Terminator Strength Prediction (Tobacco Leaf System, $\hbox {R}^2$)

Method	*A. thaliana*	*S. bicolor*	*Z. mays*	Avg.
DNABERT-2	0.703	0.583	0.684	0.657
DNABERT-S	0.712	0.570	0.673	0.652

## D Benchmarking Nucleotide Transformer Variants on Plant Genomic Tasks

The Nucleotide Transformer (Dalla-Torre et al 2023) includes various models that differ in pretraining corpora and parameter sizes and also provided the Nucleotide Transformer v2, Agronomic Nucleotide Transformer. We checked that the Agronomic Nucleotide Transformer (Mendoza-Revilla et al 2024) consistently outperformed other variants, making it the most effective baseline for plant genomic prediction tasks (all tasks except chromatin accessibility) See appendix Figure 8.

## E Experimental Hardware Configurations

Appendix Table 11 summarizes the hardware environments used for each experiment.

**Table 11:** Hardware configurations used in each experiment

Experiment	GPU Model	Memory / # of GPUs
Pretraining on Plant Genomes	NVIDIA A100-SXM	40 GB / 1
*k*-mer Tokenization Strategy (PGB)	NVIDIA V100-SXM	32 GB / 1
*k*-mer Tokenization Strategy (RNA-seq)	NVIDIA RTX A6000-PCIE	48 GB / 2
Preliminary Experiments	NVIDIA V100-SXM	32 GB / 1
DNABERT Variants (PGB)	NVIDIA RTX A6000-PCIE	48 GB / 1
NT Variants (PGB)	NVIDIA V100-PCIE	32 GB / 1

PGB: Plant Genomic Benchmark

NT: Nucleotide Transformer
